# Supplementary material for: Association between Sexual Activity during Pregnancy, Pre- and Early-Term Birth, and Vaginal Cytokine Inflammation: A Prospective Study of Black Women
Source: Healthcare (Basel). 2023 Jul 11;11(14):1995. doi: 10.3390/healthcare11141995 (PMC10379435; doi:10.3390/healthcare11141995)
Supplement: Supplementary file 1 [file healthcare-11-01995-s001.zip › healthcare-2379247-supplementary.pdf]

Supplementary Table S1 Descriptive statistics of ratios between pro- and anti- inflammatory cytokines by birth outcomes

Ratio[1]	Spontaneous Preterm Birth	Spontaneous Early Term Birth	Full-term Birth (referent)
Serum IFNg/IL10 (T1)	2.07±1.03	2.09±0.93	2.12±0.70
Serum IFNg/IL10 (T2)	1.82±1.11	1.91±0.84	1.97±0.87
Vaginal IFNg/IL10 (T1)	2.74±1.67	2.84±1.93	3.07±1.74
Vaginal IFNg/IL10 (T2)	3.09±1.66	3.50±1.56	2.81±1.89
Serum IL6/IL10 (T1)	0.42±0.73	0.66±1.01	0.76±0.86
Serum IL6/IL10 (T2)	0.42±0.90	0.75±0.96	0.74±0.88
Vaginal IL6/IL10 (T1)	3.67±2.35	3.58±2.12	3.47±2.04
Vaginal IL6/IL10 (T2)	2.75±1.70	3.43±2.11	2.73±2.16
Serum CRPmg (T1)	-1.02±1.19	-0.98±1.46	-0.84±1.28
Serum CRPmg (T2)	-0.91±1.03	-0.96±1.31	-0.61±1.07
Vaginal CRPmg (T1)	-9.32±1.84	-9.31±1.85	-9.43±2.01
Vaginal CRPmg (T2)	-9.74±1.83	-9.31±1.97	-9.30±1.95
Serum TNFa/IL10 (T1)	1.84±0.60	1.89±0.71	2.02±0.63
Serum TNFa/IL10 (T2)	1.83±0.76	1.95±0.82	2.13±0.79
Vaginal TNFa/IL10 (T1)	1.91±1.68	1.87±1.90	1.96±1.52
Vaginal TNFa/IL10 (T2)	1.48±1.69	2.07±1.94	1.65±1.57

[1] Ratios between pro- and anti- inflammatory cytokines were log-transformed to normalize the data distribution. T1 and T2 stand for early and late pregnancy, respectively.
